# Lumbar round cell sarcoma in a 10-week-old rottweiler puppy

**DOI:** 10.1186/s13028-025-00800-1

**Published:** 2025-03-12

**Authors:** Katrine Vestergaard Kristiansen, Anders Simon Schrøder, Dorothee Bienzle, Tanja Vedel, Jørgen Steen Agerholm, Mette Berendt

**Affiliations:** 1 Department of Veterinary Clinical Sciences, Faculty of Health and Medical Sciences, University of Copenhagen, Dyrlaegevej 16, Frederiksberg C, DK-1870 Denmark; 2 Department of Pathobiology, Ontario Veterinary College, University of Guelph, 50 Stone Road East, Guelph, Ontario N1G 2W1 Canada

**Keywords:** Dog, Juvenile, Neoplasia, Spinal disease, Tumour

## Abstract

**Background:**

Spinal neoplasms are sparsely documented in juvenile dogs. Case reports and small case series have described nephroblastomas, primitive neuroectodermal tumours, gliomas, certain sarcomas, and osteochondromas, but round cell sarcomas have not previously been documented.

**Case presentation:**

This case report describes a 10-week-old female Rottweiler puppy with acute onset of progressive ataxia and pelvic limb lameness. Neurological examination localised a T3-L3 myelopathy and MRI revealed an ovoid, well-marginated mass extending from mid L3 to caudal L4 vertebrae. Post-mortem examination, histopathology, and immunohistochemistry confirmed a round cell sarcoma of extradural origin.

**Conclusion:**

Our case report stresses the importance of performing MRI even in very young individuals with acute progressive signs of spinal cord lesions. Clinicians should include spinal tumours as a differential diagnosis for juvenile canines with spinal neurological signs. Round cell sarcoma should be added to the list of spinal tumours in young dogs.

## Introduction

Spinal neoplasms account for around 9% of dogs presenting with spinal cord disease [1]. The clinical onset is typically subacute-chronic and may include spinal hyperaesthesia and neurological deficits [1–3]. Spinal neoplasia is well documented in middle-aged to older, large breed dogs [1, 4–8] but sparsely documented for juvenile and young dogs. Two retrospective studies, investigating 29 and 27 dogs with spinal neoplasia, identified only three dogs aged < 7 months [9–10]. Another retrospective study investigated magnetic resonance imaging (MRI) features of extradural spinal neoplasia in 60 dogs aged five months to 13 years, but did not specify how many dogs were juvenile [2]. The same study found round cell tumours to be the second most common tumour type, and small tumours were more frequent than medium or large tumours [2]. Larger surveys on spinal tumours in young dogs are not available. Case reports or small case series include nephroblastoma [11–12], primitive neuroectodermal tumour [13–14], glioma [15], sarcoma [16–18], and osteochondroma [19–21].

Here we report on a 10-week-old Rottweiler with acute progressive pelvic limb ataxia and spinal pain. A highly destructive soft tissue mass affecting the L3-L4 vertebrae found on MRI was identified as a spinal round cell sarcoma on histopathology. Round cell sarcoma adds to the list of spinal tumours in young dogs and should be considered when an extradural tumour is observed on imaging.

## Case history

**Fig. 1:**
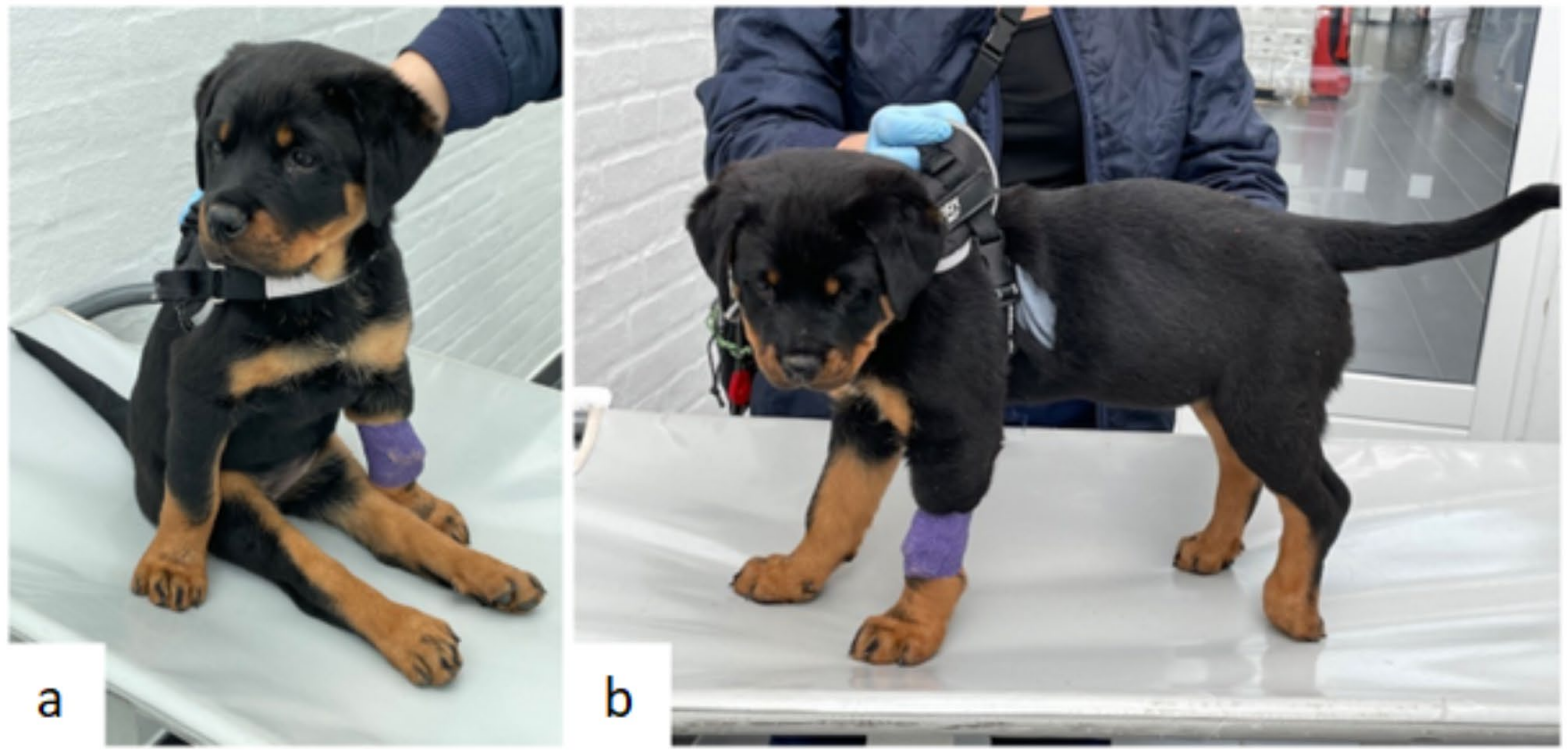
Photographs of the Rottweiler puppy during the neurological examination. On clinical presentation, the puppy presented non-ambulatory paraparetic (**a**) with decreased pelvic limb proprioceptive placing (**b**)

A 10-week-old female Rottweiler was referred with a 4-day history of progressive pelvic limb ataxia and lumbosacral pain. At presentation, the puppy was bright and alert, non-ambulatory paraparetic (Fig. 1a) but able to stand and bear some weight if supported. There was marked pain response to palpation of the lumbar region. Cranial nerve tests were normal. Proprioceptive placing was decreased for both pelvic limbs, more severely on the left (Fig. 1b). All spinal reflexes were normal, and the puppy was able to urinate and defecate spontaneously. A T3-L3 spinal cord lesion was suspected. Haematology showed mild anaemia and mild monocytosis. Biochemistry was unremarkable. The initial differential diagnoses comprised fracture, haemorrhage, neoplasia, congenital malformation, and discospondylitis. Due to pain originating from the lumbosacral region, radiographs of this region were taken to rule out a spinal fracture before performing MRI. Radiographs were unremarkable.

A high-field MRI-system with a spine array coil (1.5T Siemens Magnetom Altea, Siemens Healthcare A/S, Ballerup, Denmark) was used to obtain spinal images from T3 to S1 using a T2-weighted turbo spin echo (TSE) sagittal sequence resulting in lesion identification in the L3-L4 region. Subsequent imaging focused on T10 to S1 including T1-weighted, T2-weighted, short tau inversion recovery (STIR), T1-weighted fat-saturated, and T1-weighted post-contrast multiplanar sequences.

**Fig. 2:**
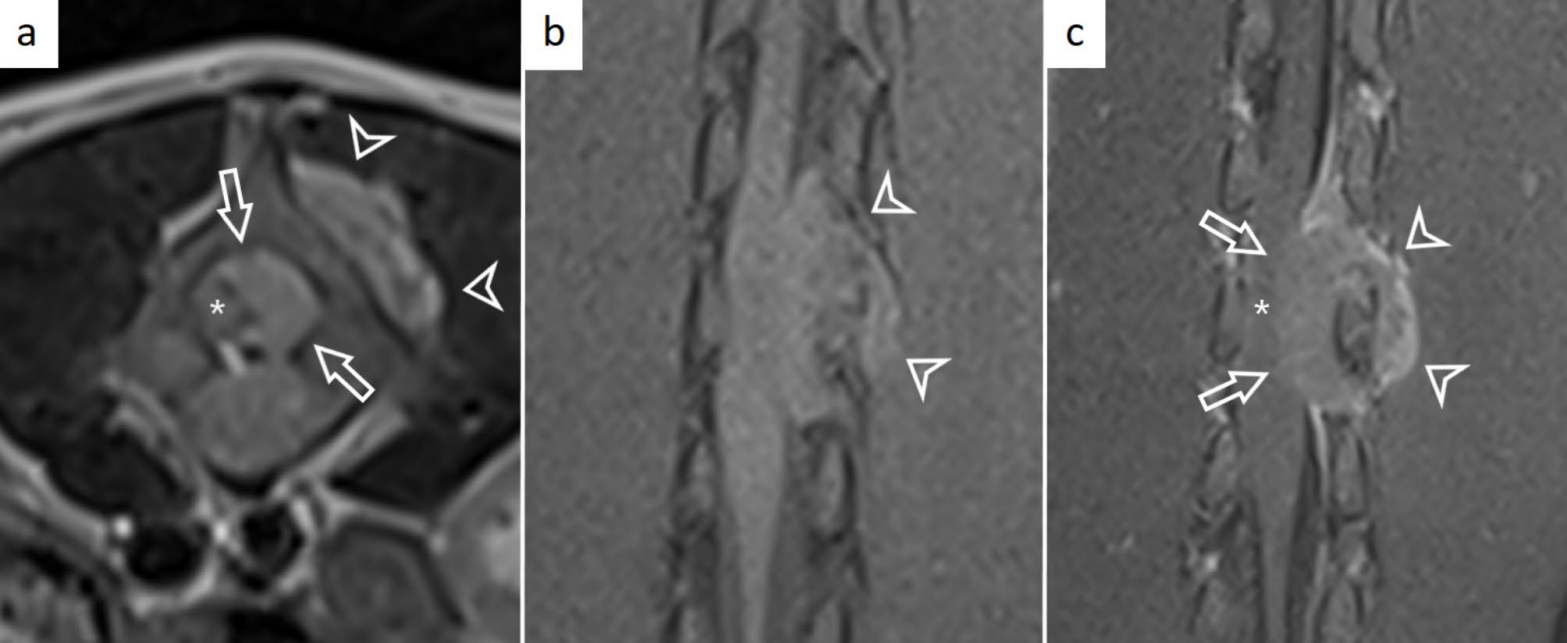
Magnetic resonance imaging findings of the lumbar spine. Transverse T2-weighted image at the level of the L4 vertebra (**a**) and dorsal T1-weighted image on the level of the spinal cord pre-contrast (**b**) and post gadolinium contrast (**c**) showing a confirmed extradural mass encompassing the left pedicle of L4. The mass appears hyperintense on T2-weighted sequences (**a**), isointense to the spinal cord on T1-weighted sequences (**b**), and moderately, heterogeneously contrast enhancing with meningeal enhancement “dural-tail”-sign (**c**). The mass extends into the vertebral canal (white arrows) and left dorsolateral (white arrowheads). The mass displaces and compresses the spinal cord towards the right (asterisk)

An ovoid, well-marginated lesion exerting mass effect extending from the mid L3 vertebral body to the caudal L4 vertebra was identified. The mass was centred at the left pedicle of L4, extending into the ipsilateral vertebral foramen and dorsolaterally just left of the L4 vertebra. The mass was homogeneously hyperintense on T2-weighted sequences and heterogeneously iso-to-hyperintense on T1-weighted sequences compared to normal spinal cord (Fig. 2). The mass seemed to infiltrate the right dorsal aspect of the vertebral arch and pedicle of L4, with decreased signal intensity of the bone. The mass measured 2.8 cm x 1.3 cm x 1.1 cm (length x width x height) and occupied approximately 75% of the cross-sectional area of the vertebral canal at maximum, causing focal rightward displacement and moderate compression of the spinal cord (Fig. 2). Subsequent widening of the subarachnoid space cranially and caudally was evident. The mass had a para-vertebral extension across the left-intervertebral foramina of L3-L5. There was moderate heterogeneous contrast enhancement, most pronounced in the para-spinal tissues in post-gadolinium T1-weighted sequences. Meningeal enhancement was seen cranially and caudally to the mass within the vertebral canal (Fig. 2). Localisation of the mass based on imaging was primarily intradural-extramedullary with an extradural component. The imaging diagnosis was soft tissue neoplasia with osseous invasion.

**Fig. 3:**
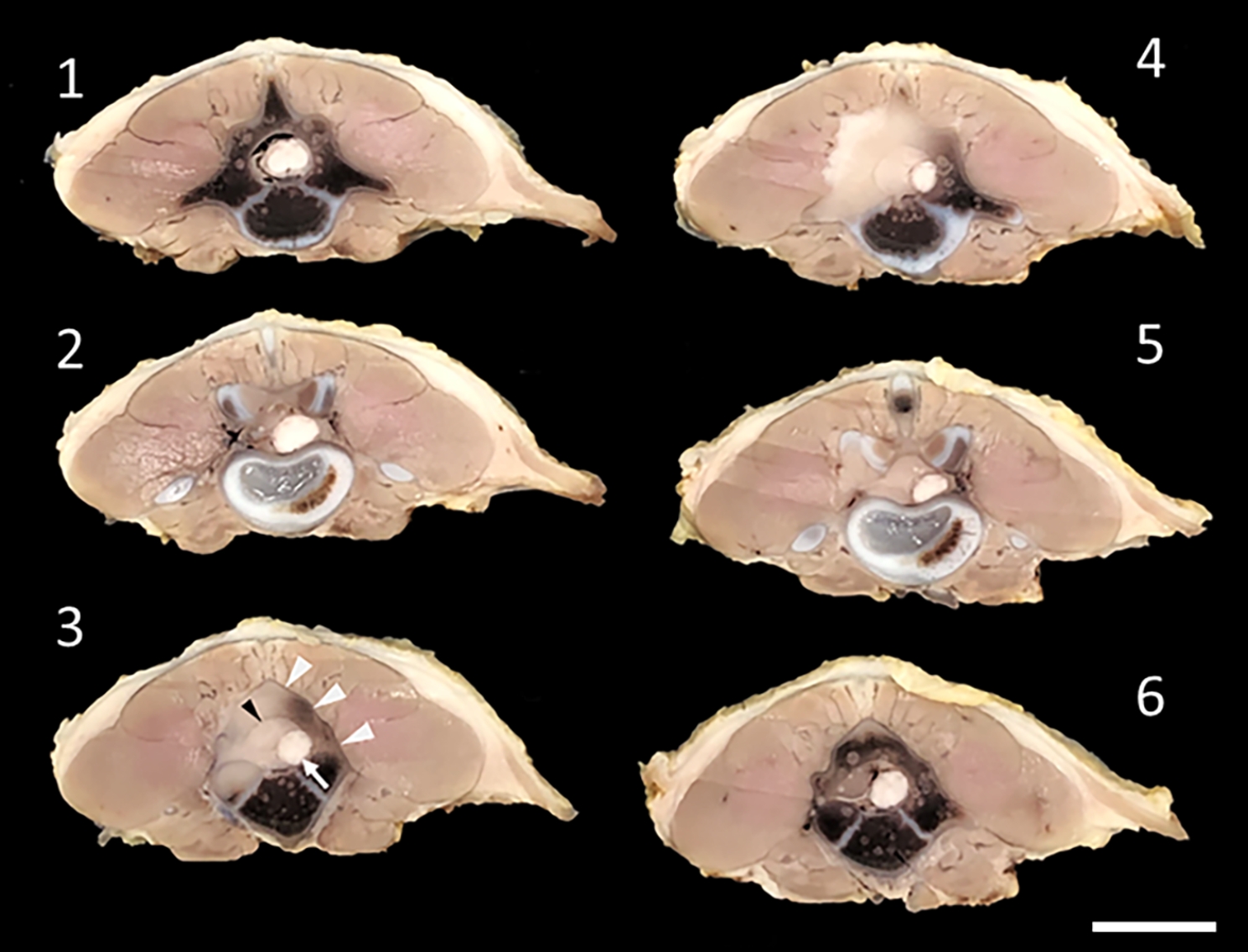
Necropsy findings of the lumbar spine. Serial 4 mm transverse sections through the lumbar region in cranial (**1**) to caudal (**6**) direction. A pale soft tissue mass is in the vertebral canal and causes dislocation of the spinal cord (exemplified in specimen 3, white arrow). The outline of the vertebral canal is maintained (specimen 3, black arrowhead). The mass expands dorsolaterally, mainly to the left side and is associated with destruction of the vertebral arch. The outline of the arch is mostly maintained (specimen 3, white arrow heads). Notice the distinct border towards the surrounding muscles. Formalin fixed and decalcified specimen. Bar = 2 cm

As the mass was extensive and involving bone structures, the puppy was euthanized due to a poor prognosis. Necropsy findings were unremarkable except for the lumbar lesion. The entire lumbar region was fixed in 10% neutral buffered formalin before being decalcified in a 15% formic acid solution. Subsequent examination of transverse 4 mm serial sections revealed an epidural pale uniform soft tissue mass at L3-L4 that dislocated the spinal cord and filled the vertebral canal. The L3 vertebral arch, especially to the left, was almost completely missing and replaced by the soft tissue mass. The mass had expanded dorsolaterally causing atrophy of the deep parts of the longissimus dorsi muscle. The mass was well-delineated in most areas, and the contour of the spinal canal and vertebral arch had mostly remained (Fig. 3).

**Fig. 4:**
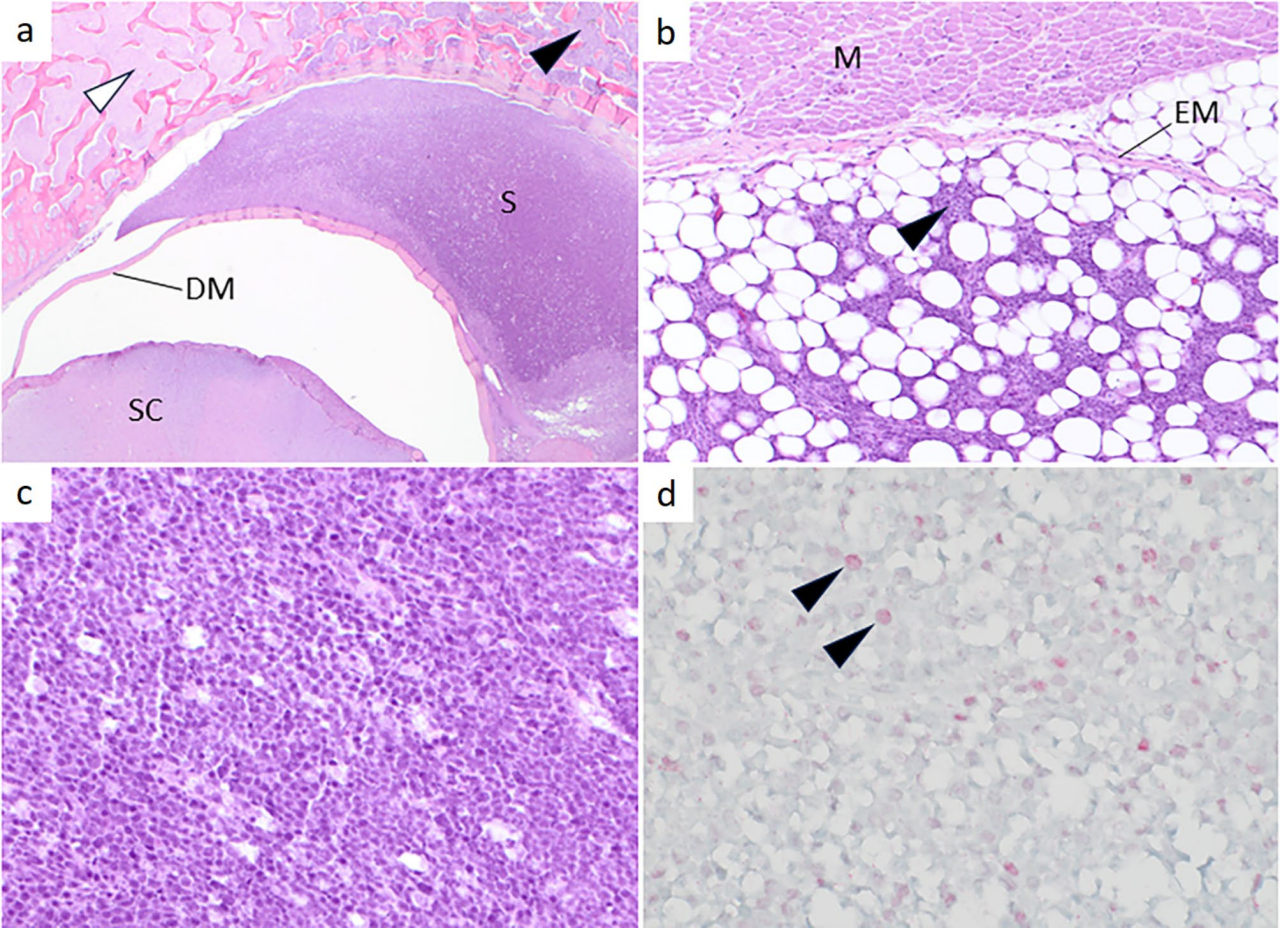
Histological and immunohistochemical features of a lumbar round cell sarcoma. (**a**) The extradural localization of the sarcoma (S) is evident. Neither the spinal cord (SC) nor the dura mater (DM) is infiltrated by tumour cells, but the tumour has expanded peripherally, and neoplastic cells have completely replaced the bone marrow in some locations (black arrowhead). Non-neoplastic bone marrow is indicated by a white arrowhead. (**b**) Adipose tissue located between the vertebral bone and the longissimus dorsi muscle (M) is infiltrated by neoplastic cells (black arrowhead), but an intact epimysium persists as a barrier to neoplastic cells infiltrating into the muscle. (**c**) Higher magnification showing a tumour composed of a uniform population of pleomorphic round to slightly angular cells with a scant amount of basophilic cytoplasm and absence of a matrix. (**d**) Immunohistochemical staining for the proliferation marker Ki-67. Approximately 14% of cells have pink (positive) immunostaining in the nucleus (black arrowhead). **a**-**c**: Haematoxylin and eosin. **d**: Fast red chromogen and haematoxylin

Histologically, the neoplasm was considered to originate at an extradural location, and to cross the periosteum and expand into vertebral trabecular bone, where regions were necrotic. The neoplasm consisted of sheets of individual pleomorphic round to slightly angular cells. Neither tubule nor rosette formation were apparent, and the tumour cells were not embedded in matrix (Fig. 4). Cells were of medium to large size relative to endothelial cells, and had a scant amount of basophilic cytoplasm, nuclear to cytoplasmic ratio of > 3:1, and frequent but variably numerous and variably sized nucleoli. Some nucleoli, when single, were extremely large (up to 5-fold the size of endothelial cell nuclei). There was an average of 29 mitotic figures per ten fields at 400× magnification (0.237 mm^2^). The brain, other spinal cord segments and a range of other tissues were unremarkable.

Multiple immunohistochemical assays were applied to tumour sections (Table 1). Reactions were interpreted in relation to non-neoplastic tissue included in tumour sections (i.e. nerve cross-sections, endothelium) and in relation to reactions with positive and negative control sections consisting of multiple canine tissues. Negative controls consisted of assays with omission of the primary antibody. All immunohistochemical reactions had been validated for canine tissue in accordance with guidelines of the American Association of Veterinary Laboratory Diagnosticians at the Animal Health Laboratory (AHL), University of Guelph, Canada. The neoplastic cells were negative for all IHC reactions except approximately 14% staining positive for the proliferation marker Ki-67 (Fig. 4). Based on histomorphology and immunohistochemical staining pattern, the neoplasm was diagnosed as a poorly differentiated round cell sarcoma.

**Table 1:** Summary of immunohistochemical protocols used on tissue sections

Cell marker	Antibody type	Host	Source	Antigen retrieval^a^	Dilution	Chromogen
Vimentin	Monoclonal	Mouse	Dako	pH 8	1:64	Ultraview ALP Red^b^
Cytokeratin AE-1/AE-3	Monoclonal	Mouse	Dako	pH 9	1:200	Nova Red^c^
Iba1	Polyclonal	Rabbit	Wako	pH 8	1:2,000	Ultraview ALP Red
GFAP^d^	Polyclonal	Rabbit	Dako	PK^e^	1:50	Nova Red
S100	Monoclonal	Mouse	Dako	pH 8	1:2,000	Ultraview ALP Red
Ki-67	Polyclonal	Rabbit	Intermedico	pH 8	1:100	Ultraview ALP Red
NSE^e^	Polyclonal	Rabbit	Abcam	pH 6	1:50	Nova Red
OLIG2^f^	Monoclonal	Rabbit	Sigma	pH 6	1:150	Nova Red
Pax5	Polyclonal	Rabbit	Biocare	pH 9	1:50	Nova Red
CD3	Polyclonal	Rabbit	Dako	pH 6	1:100	Nova Red
CD18	Monoclonal	Mouse	UC Davis	pH 6	1:40	Nova Red

^a^ Heat-induced antigen retrieval; ^b^ Ventana/Roche; ^c^ Vector Labs; ^d^ glial fibrillary acidic protein; ^e^ neuron specific enolase; ^f^ oligodendrocyte transcription factor 2

## Discussion and conclusions

This is the first report of a spinal round cell sarcoma in a juvenile dog.

In humans, round cell sarcomas are typically high-grade tumours that may be challenging to diagnose due to poor differentiation [22]. The most common round cell sarcoma, Ewing sarcoma, is the second most prevalent sarcoma in children, and can even be seen in infancy [23–24]. Around 80% originate from bone and 20% from soft tissue [22]. According to the 2020 WHO Classification of Soft Tissue Tumours “the differential diagnosis for these tumours is rather broad, and among round cell sarcomas includes alveolar rhabdomyosarcoma, desmoplastic small round cell tumour, poorly differentiated round cell synovial sarcoma, small cell osteosarcoma, and mesenchymal chondrosarcoma” [25].

Despite the initial suspicion of an intradural tumour localisation on MRI, post-mortem examination revealed an extradural localisation. Discrepancies between imaging localisation and actual tumour localisation has recently been investigated in human medicine, where it was found that tumours invading multiple compartments were most often mis-localised by radiologists. Extradural tumours were also sometimes mis-localised as intradural [26].

Clinical signs were observed by the owners 4 days prior to presentation at the age of 10 weeks. At that time, tumour growth was extensive with peripheral expansion and massive loss of vertebral bone (Fig. 3). Despite the extensive bone loss, infiltrative growth was limited to invasion of hematopoietic tissue in between trabecular bone. Tumour cells did not infiltrate the dura mater and the margin towards the surrounding muscles was distinct. Extensive loss of bone as seen in the present case requires more than a few days to develop, but the onset of tumorigenesis could not be determined.

Although rarely reported, neoplasia must be considered when young individuals develop spinal neurological signs, although other causes such as trauma, congenital malformations, and inflammatory disease are more likely. Several degenerative neurological and neuromuscular diseases and syndromes have also been recorded for young Rottweilers [27] but none of these fit with the clinical presentation of the present case. Our case report stresses the importance of performing MRI for acute progressive myelopathies even in juvenile individuals. Aiming for early detection can prevent unnecessary suffering in cases where the prognosis is poor or help guide treatment options when possible.

## Data Availability

No datasets were generated or analyzed during the current study. Results from diagnostic modalities are available from the corresponding author on reasonable request.

## Declarations

### Prior publication

Data have not been published previously.
